# A role for the purinergic receptor P2X_3_ in astrocytes in the mechanism of craniofacial neuropathic pain

**DOI:** 10.1038/s41598-017-13561-3

**Published:** 2017-10-19

**Authors:** Won Mah, Sang Man Lee, Jaekwang Lee, Jin Young Bae, Jin Sook Ju, C. Justin Lee, Dong Kuk Ahn, Yong Chul Bae

**Affiliations:** 1 Department of Anatomy and Neurobiology, School of Dentistry, Kyungpook National University, Daegu, 700-412 Korea; 2 Center for Neuroscience and Functional Connectomics, Brain Science Institute, Korea Institute of Science and Technology (KIST), Seoul, 02791 Korea; 3 Department of Oral Physiology, School of Dentistry, Kyungpook National University, Daegu, 700-412 Korea

## Abstract

The purinergic receptor P2X_3_, expressed in the central terminals of primary nociceptive neurons in the brainstem, plays an important role in pathological pain. However, little is known about expression of P2X_3_ in the brainstem astrocytes and its involvement in craniofacial pathologic pain. To address this issue, we investigated the expression of P2X_3_ in astrocytes in the trigeminal caudal nucleus (Vc) in a rat model of craniofacial neuropathic pain, chronic constriction injury of infraorbital nerve (CCI-ION). We found that 1) P2X_3_-immunoreactivity is observed in the brainstem astrocytes, preferentially in their fine processes, 2) the number of P2X_3_-positive fine astrocytic processes and the density of P2X_3_ in these processes were increased significantly in CCI-ION rats, compared to control rats, and 3) administration of MPEP, a specific mGluR5 antagonist, alleviated the mechanical allodynia and abolished the increase in density of P2X_3_ in fine astrocytic processes caused by CCI-ION. These findings reveal preferential expression of P2X_3_ in the fine astrocytic processes in the brainstem, propose a novel role of P2X_3_ in the fine astrocytic process in the mechanism of craniofacial neuropathic pain, and suggest that the expression of astrocytic P2X_3_ may be regulated by astrocytic mGluR5.

## Introduction

The purinergic receptor P2X_3_ is a nonselective cation channel, sensitive to ATP released during injury^1–3^. Inhibition of P2X_3_ in the brainstem or the spinal dorsal horn reduces hypersensitivity following nerve injury, suggesting that P2X_3_ plays a role in pathological pain^4–6^. This effect is believed to be mediated by primary nociceptive neurons since P2X_3_ is expressed in their central terminals^7–10^. However, little is known about the expression of P2X_3_ in the astrocyte in the brainstem or spinal cord, and its possible involvement in pathologic pain.

Astrocytes are implicated in pathological pain associated with nerve injury and inflammation^11–14^. Many studies suggested that fine astrocytic processes may be involved in the regulation of neuronal activity and synaptic transmission^15–18^. In addition, under physiological condition, astrocytic fine processes undergo profound morphological changes (retraction and protrusion) that modify their relationship with synapses and adjacent neurons^19–21^. In contrast, under pathological conditions, including nerve injury and inflammation, most studies in the brainstem and spinal cord describe hypertrophy and GFAP upregulation in astrocytic soma and large processes that may not be directly involved in the regulation of neuronal activity as distinctive morphological features of reactive astrocytes^13,14,22^. So far, however, how astrocytes related to nearby neurons may change their spatial relationship with synapse, following nerve injury, remains poorly understood, at least from a morphological standpoint.

In this study, to address these issues, we investigated the expression of P2X_3_ in the soma, large and fine process compartment of astrocyte in the trigeminal caudal nucleus (Vc) and its involvement in the mechanism of pathologic pain in a rat model of craniofacial neuropathic pain, chronic constriction injury of infraorbital nerve (CCI-ION). We also tested the hypothesis that the activity of P2X_3_ in astrocytes is regulated via mGluR5 signaling.

## Results

### Astrocytes in the Vc express P2X_3_ receptors

**Figure 1:**
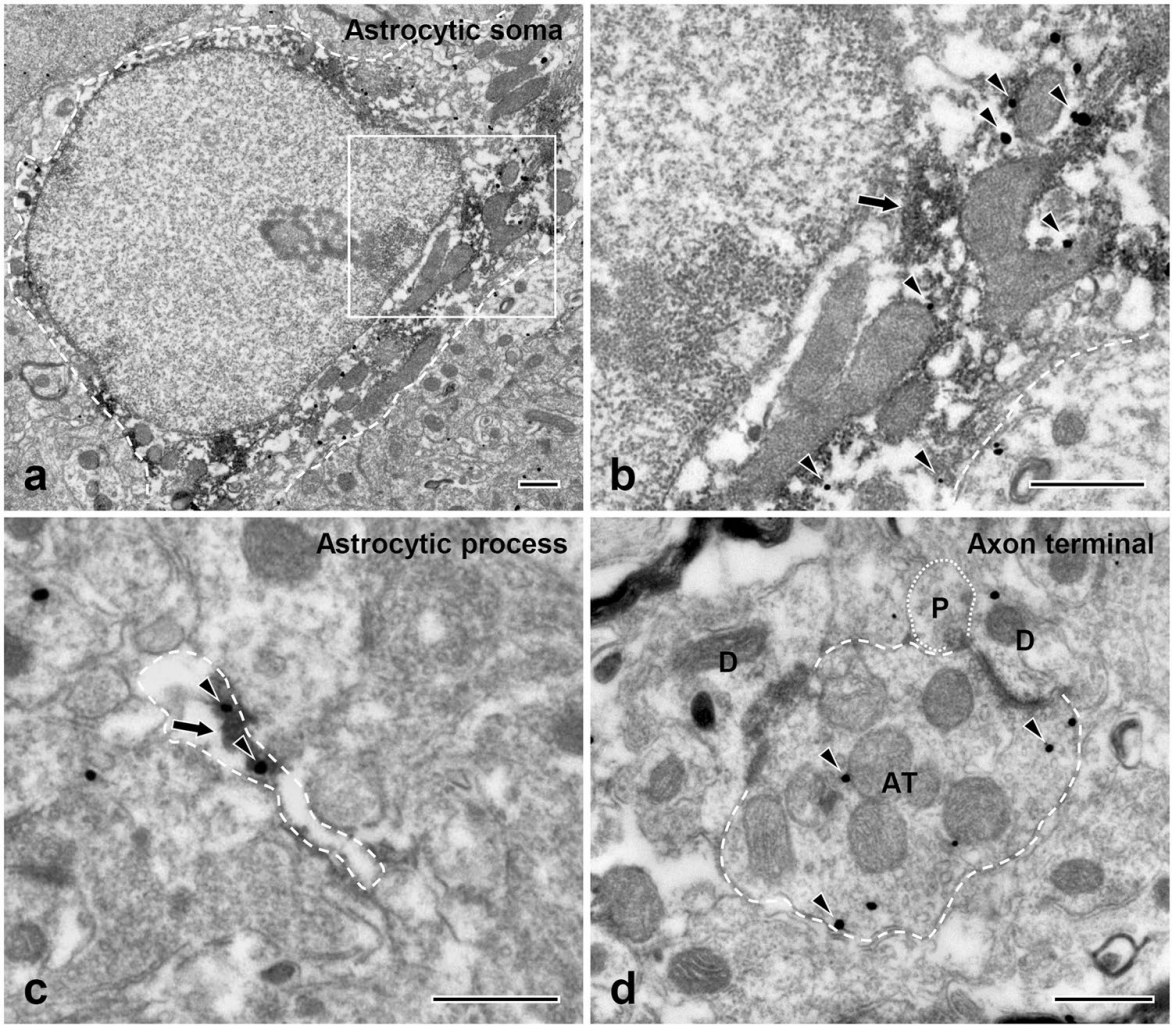
Electron micrographs showing P2X_3_ expression (silver-gold labeling, discrete, mostly round black particles) in the soma (**a**,**b**) and process (**c**) of GFAP+ astrocyte (peroxidase labeling, diffuse amorphous darker deposits) and in an axon terminal (AT: **d**) of presumed primary afferent origin in the Vc of a control rat. B is an enlargement of the boxed area in panel A. The axon terminal (AT, **d**) shows features characteristic of a primary afferent terminal: round vesicles, asymmetric contact with a dendrite (D), and axoaxonic synapse with another terminal, containing pleomorphic vesicles (P). Arrows indicate immunperoxidase labeling for GFAP. Arrowheads indicate silver-gold labeling for P2X_3_. Astrocytic soma, astrocytic process, and axon terminal are outlined by a dashed line; terminal containing pleomorphic vesicles is outlined by a dotted line. Scale bars = 500 nm.

At the electron microscopic (EM) level, the immunostaining for P2X_3_ in the superficial lamina of the Vc was identified by discrete silver-gold particles, easily distinguishable from that for GFAP, which was in the form of amorphous, electron-dense patches of reaction product (Fig. 1). Immunostaining for P2X_3_ was observed in the somata and processes of GFAP-immunopositive (GFAP+) astrocytes (Fig. 1a–c) and axon terminals (Fig. 1d) with ultrastructural features characteristic of primary afferents: round vesicles, asymmetric synapses with small dendrites, and frequent axoaxonic synapses.

### CCI-ION induces mechanical allodynia and an increase in the number of fine astrocytic processes

**Figure 2:**
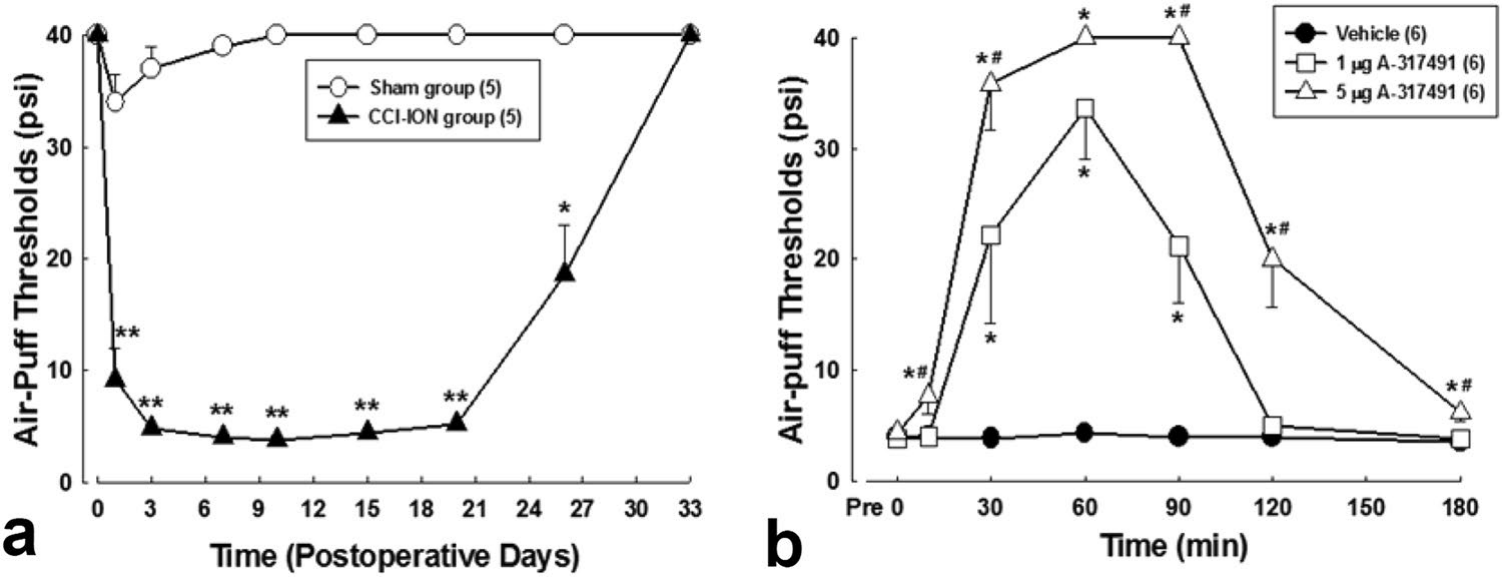
The effects of A-317491, a specific antagonist of P2X_2_ and P2X_3_ receptors, on mechanical allodynia in CCI-ION rats. (**a**) Time-course analysis of changes in air-puff thresholds after nerve injury. CCI-ION produced significant decreases in air puff-thresholds compared to sham group (*P < 0.05, **P < 0.01, sham *vs*. CCI-ION group). N = 5 animals per group. (**b**) Intracisternal administration of A-317491 produced anti-allodynic effects in a dose dependent manner (repeated-measures analysis of variance, followed by Holm-Sidak post-hoc analysis; *P < 0.05, *^#^ *P* < 0.01, vehicle *vs*. A-317491-treated group). N = 6 animals per group.

Rats with CCI-ION manifested obvious nociceptive behavior: Air puff-thresholds were significantly lower than control. Mechanical allodynia presented on postoperative day 1 and persisted until 27 days after surgery (Fig. 2a). Intracisternal administration of A-317491, a specific antagonist of P2X_2_ and P2X_3_ receptors, produced significant anti-allodynic effects in a dose-dependent manner, compared to intracisternal administration of vehicle (Fig. 2b).

**Figure 3:**
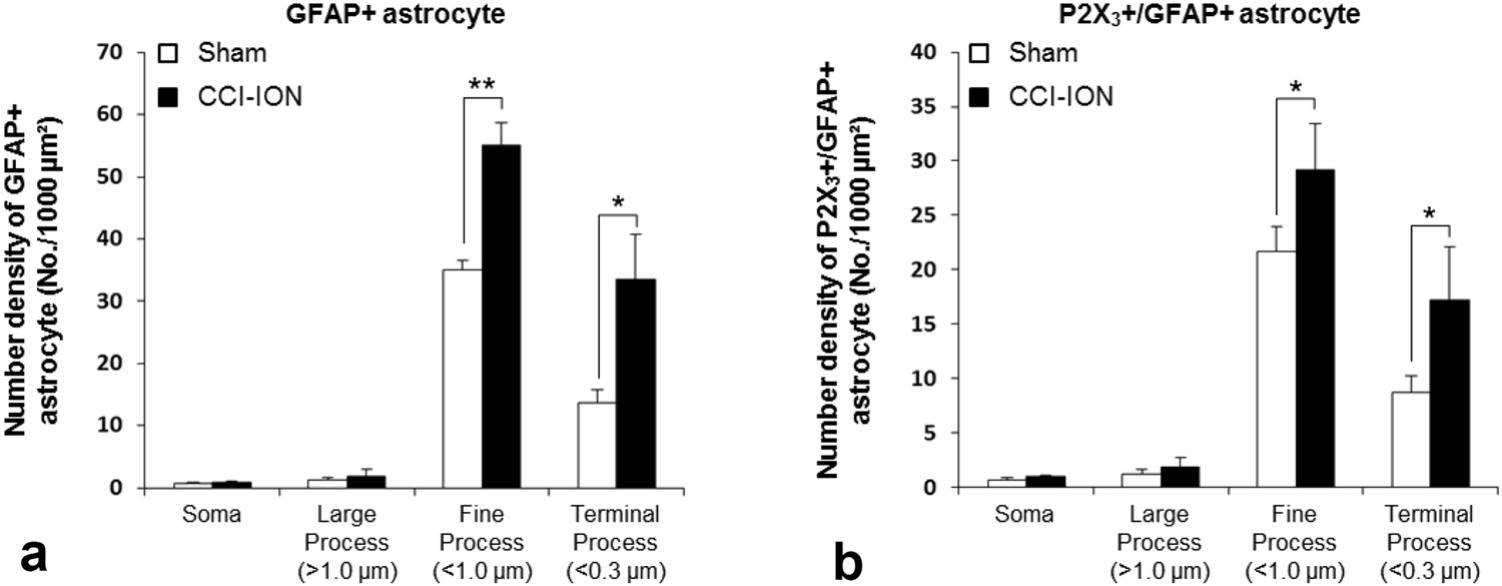
Histogram showing density (number of GFAP+ and of P2X_3_+/GFAP+ astrocyte per 1,000 μm^2^ area) of astrocytic soma, large (>1 μm in diameter), fine (<1 μm) and terminal (<0.3 μm) processes in the superficial lamina of Vc in the CCI-ION and sham rats. The density of the GFAP+ and P2X_3_+/GFAP+ is significantly increased in the CCI-ION rats, compared to sham rats in fine (1.3–1.6 times) and terminal astrocytic processes (2.0–2.4 times). Asterisks indicate significant difference between CCI-ION and sham rats (unpaired Student’s *t*-test; *P < 0.05, **P < 0.01). N = 3 animals per group.

At EM, the density (number per 1,000 μm^2^) of GFAP+ and P2X_3_+/GFAP+ fine astrocytic process in the superficial lamina of the Vc was significantly higher in CCI-ION rats than in control rats (Fig. 3). This increase in density after CCI-ION appeared to be inversely correlated to the size of the astrocytic process, it was 2.0–2.4 times for terminal processes (<0.3 μm in diameter), 1.3–1.6 times for fine processes (<1 μm in diameter), and there was no detectable difference in the density of astrocytic soma and large processes (>1 μm in diameter) between CCI-ION rats and controls (Fig. 3).

### P2X_3_ is upregulated in astrocytic processes following CCI-ION

**Figure 4:**
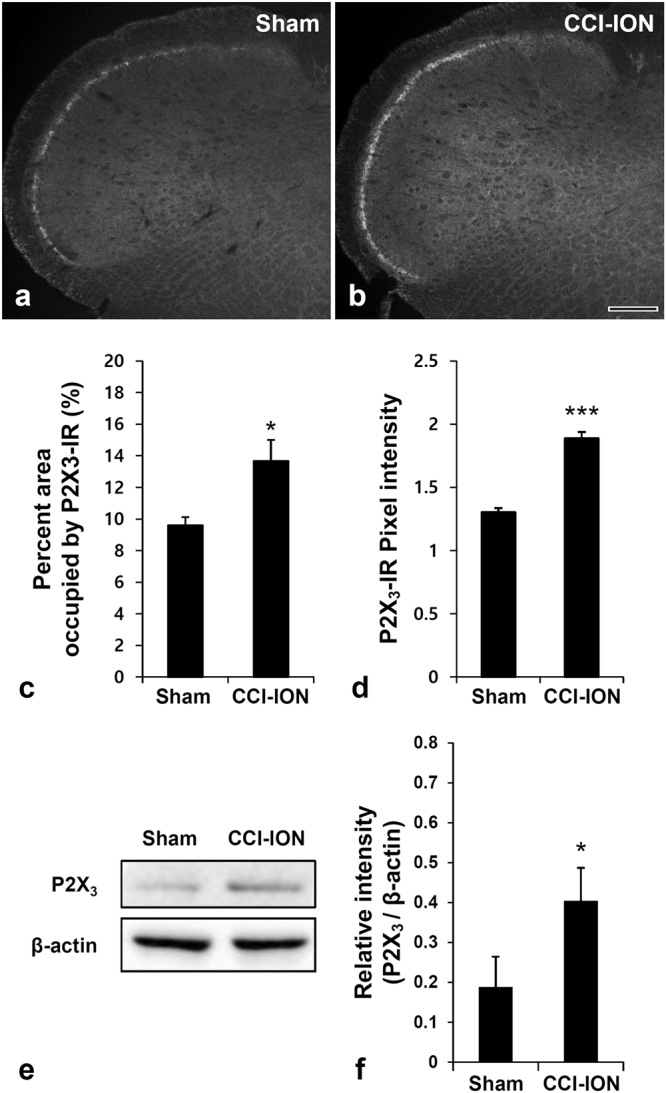
Immunofluorescent staining for P2X_3_ (**a**,**b**), area occupied by P2X_3_-immunoreactivity (IR: **c**), intensity of P2X_3_-IR (**d**) and Western blot analysis for P2X_3_ protein level (**e**,**f**) in CCI-ION rats and sham rats. The area of immunostaining for P2X_3_, the intensity of P2X_3_-IR, and the P2X_3_ protein levels are increased significantly in the CCI-ION rats, compared to sham rats. Asterisks indicate significant difference between CCI-ION rats and sham rats (unpaired Student’s *t*-test; *P < 0.05, ***P < 0.001). Scale bar in B = 50 μm. N = 3 animals per group in immunohistochemical analysis (**a**–**d**). N = 4 animals per group in Western blot analysis (**e**,**f**).

Expression of P2X_3_ in the superficial lamina of Vc was increased significantly in CCI-ION rats, compared to control, both in terms of the intensity of light microscopic (LM) immunofluorescent staining (Fig. 4a–d), and amount of protein detectable by Western blot (Fig. 4e,f).

**Figure 5:**
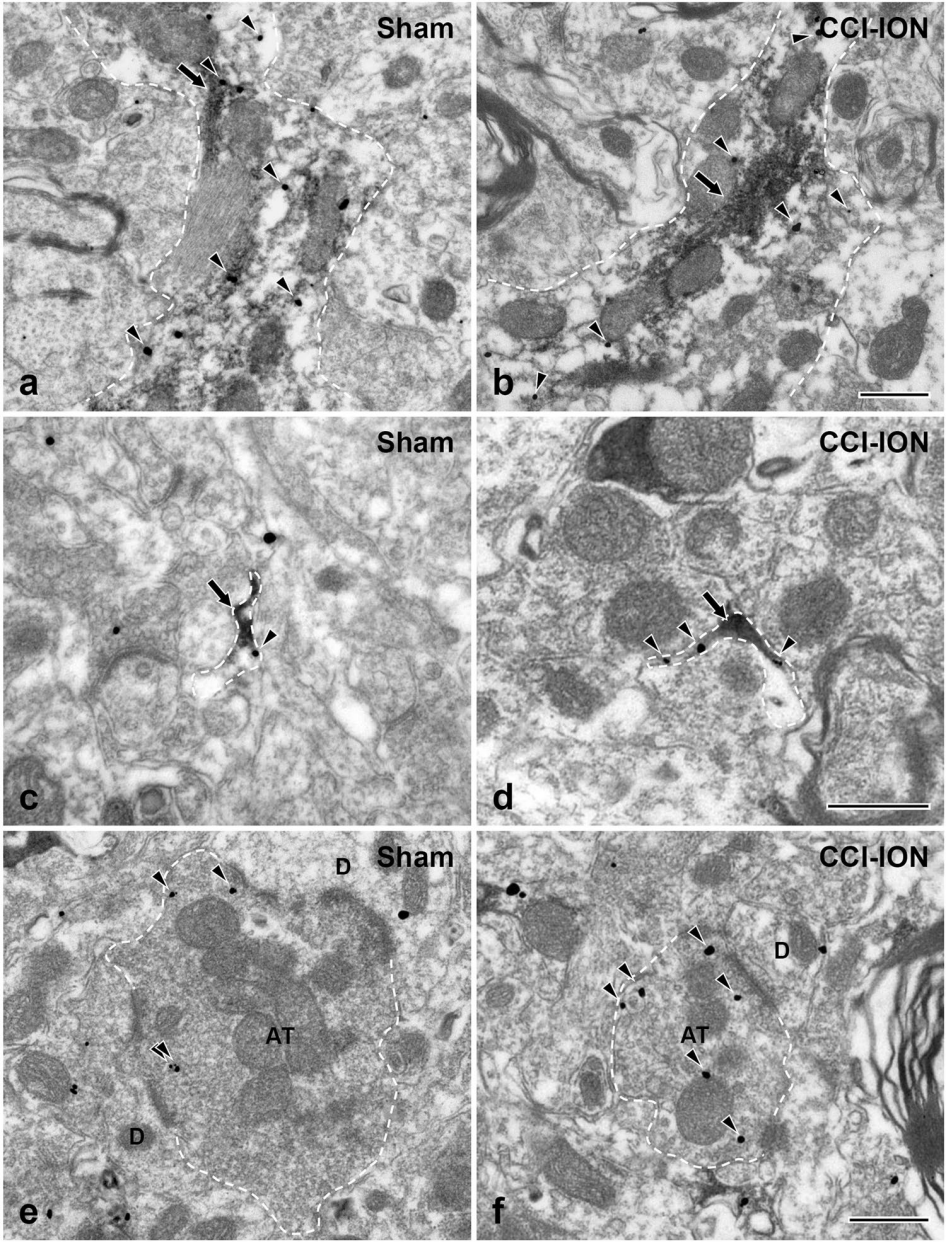
Electron micrographs showing immunolabeling for P2X_3_ (silver-gold labeling) in the GFAP+ (peroxidase labeling) large astrocytic processes (**a**,**b**) fine astrocytic processes (**c**,**d**), and axon terminal (AT; **e**,**f**) of the sham (**a**,**c**,**e**) and CCI-ION (**b**,**d**,**f**) rats. The density of P2X_3_ in fine astrocytic processes and axon terminals is higher in the CCI-ION rats than in sham rats. The axon terminal makes a synaptic contact with a small-sized dendrite (D). The astrocytic processes and the axon terminal are outlined with a dashed line. Arrowheads indicate silver-gold staining for P2X_3_. Arrows indicate immunoperoxidase staining for GFAP. Scale bar = 500 nm.

**Figure 6:**
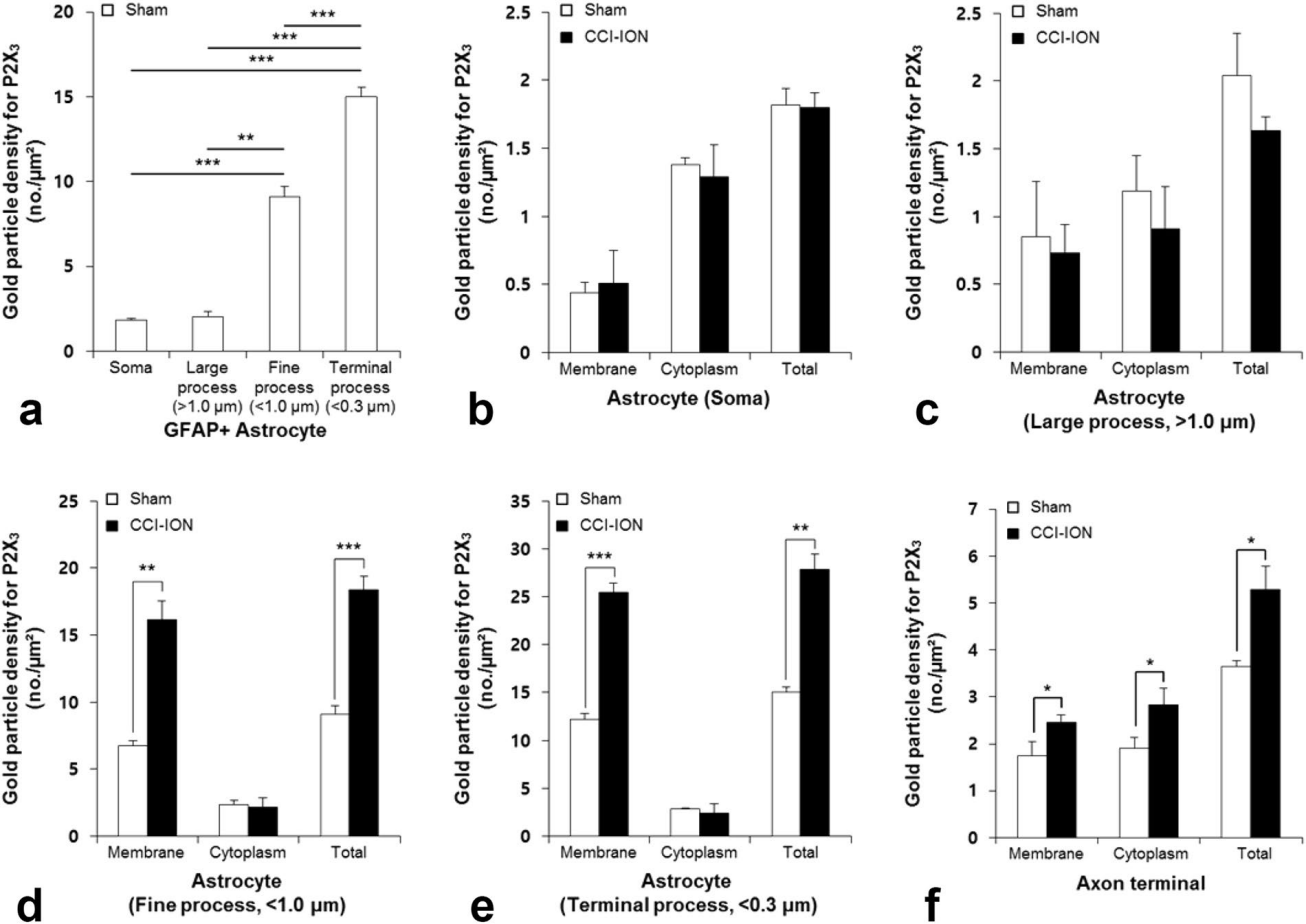
Histogram showing gold particle density for P2X_3_ in the astrocytic soma (**a**,**b**), large (**a**,**c**), fine (**a**,**d**), terminal (**a**,**e**) astrocytic processes, and axon terminals (**f**) of the sham and CCI-ION rats. In the sham rats, the density of P2X_3_ is much higher in the fine and terminal astrocytic processes than in the soma and large astrocytic processes (**a**). The density of P2X_3_ (total and associated with the plasma membrane) in the fine and terminal astrocytic processes is significantly higher in CCI-ION than in sham rats (**d**,**e**), whereas it is low in soma and large astrocytic processes, and is not significantly different between sham and CCI-ION rats (**b**,**c**). The density of P2X_3_ in axon terminals is significantly higher in CCI-ION than in sham rats. Asterisks indicate significant difference between CCI-ION rats and sham rats (unpaired Student’s *t*-test; *P < 0.05, **P < 0.01, ***P < 0.001). N = 3 animals per group.

To determine the level of expression of P2X_3_ in astrocytes and in axon terminals, we used the density of immunolabeling with silver-gold as a proxy. The density of gold particles coding for P2X_3_ was significantly higher in the fine and terminal processes than in the somata and large-caliber processes of astrocytes of normal rats (Figs 5, 6a). It was significantly increased in the fine and terminal processes in CCI-ION rats, compared to control, particularly at or immediately “under” the plasma membrane (Figs 5 and 6b–e). The density of gold particles in somata and large processes of astrocytes was not significantly different between the two groups (Figs 5 and 6b,c). These findings are consistent with upregulation of P2X_3_ in the fine astrocytic processed by CCI-ION, specifically at the membrane of astrocytic processes, presumably reflecting an increase in the functional receptor pool. Similarly, the density of gold particles in axon terminals was significantly higher in the CCI-ION rats, compared to control (Figs 5e,f and 6f).

### MPEP alleviates mechanical allodynia and abolishes the increase of P2X_3_ expression in astrocytes following CCI-ION

**Figure 7:**
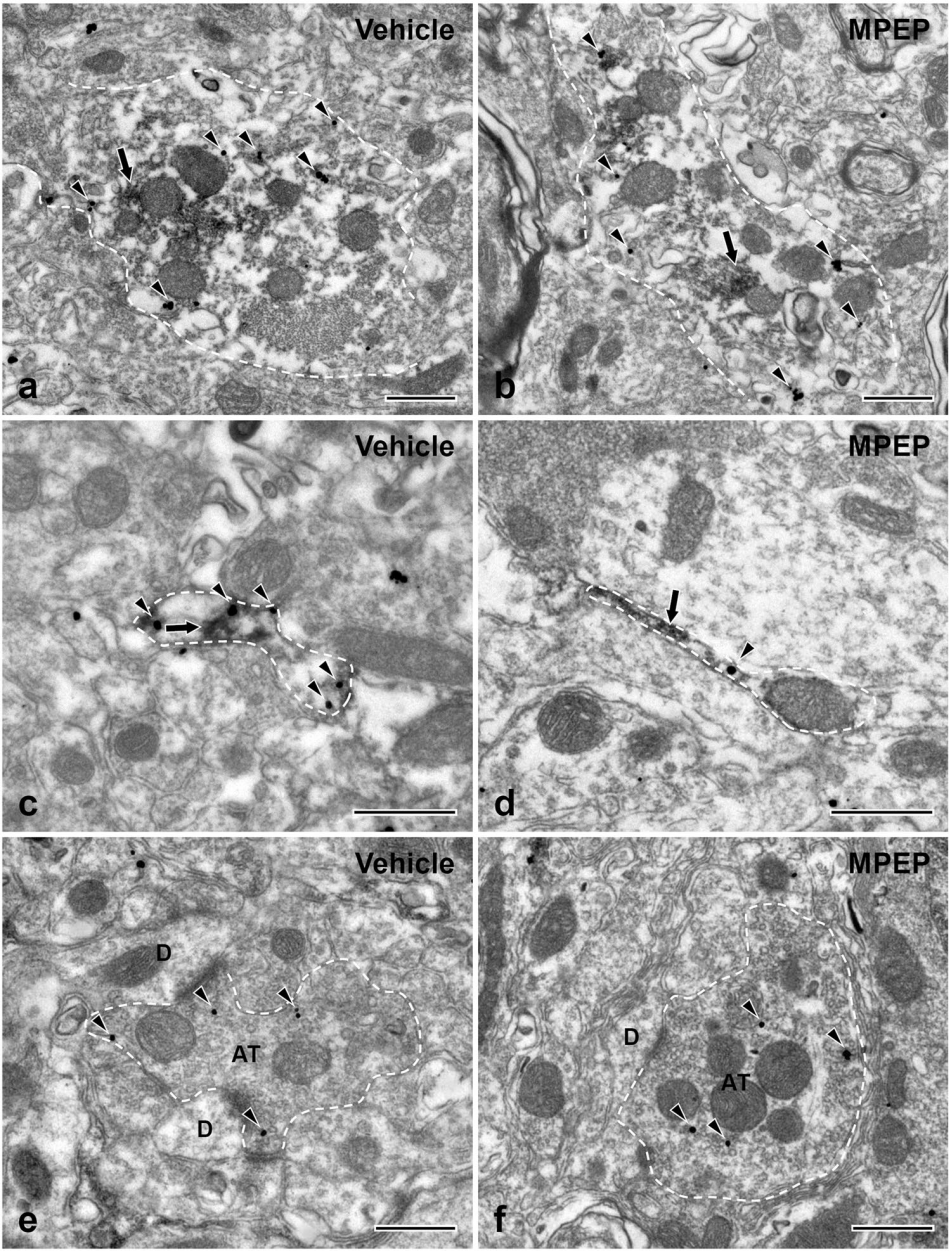
Electron micrographs showing immunolabeling for P2X_3_ (silver-gold labeling) in GFAP+ (peroxidase labeling) large (**a**,**b**) and fine astrocytic processes (**c**,**d**) and axon terminals (AT; **e**,**f**) of the CCI-ION rats following treatment with vehicle (**a**,**c**,**e**) or MPEP (**b**,**d**,**f**). The density of P2X_3_ in fine astrocytic processes is lower in the MPEP than the vehicle group (**c**,**d**), whereas that in large astrocytic processes (**a**,**b**) and axon terminals (**e**,**f**) is not significantly different between the two groups. Axon terminals (AT) make synaptic contacts with small-sized dendrites (D). The astrocytic processes and axon terminals are outlined with a dashed line. Arrowheads indicate silver-gold staining for P2X_3_. Arrows indicate immunoperoxidase staining for GFAP. Scale bar = 500 nm.

**Figure 8:**
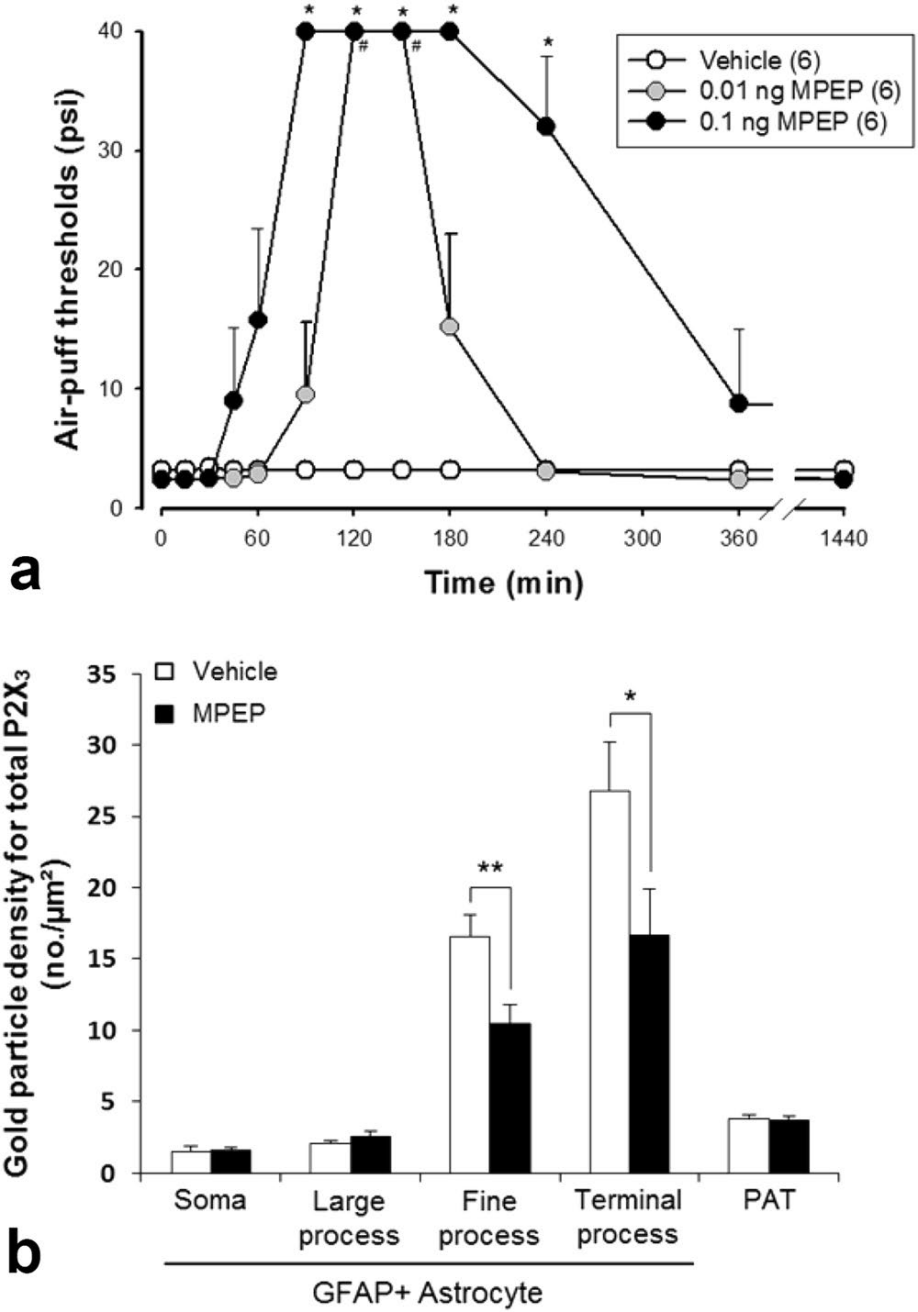
Behavioral data showing anti-allodynic effect after intracisternal administration of MPEP in CCI-ION rats (**a**) and density of P2X_3_ in astrocytic soma, fine, and terminal processes, and axon terminals following MPEP administration on post-operative day 7 in sham and CCI-ION rats (**b**). (**a**) Intracisternal administration of MPEP reversed mechanical allodynia produced by CCI-ION (*P < 0.05, vehicle *vs*. MPEP-treated group). N = 6 animals per group. (**b**) The density of P2X_3_ in fine and terminal astrocytic processes in CCI-ION rats is significantly decreased following MPEP administration, whereas that in astrocytic soma and large processes, and in axon terminals, is not different between MPEP and control groups (repeated-measures analysis of variance, followed by Holm-Sidak post-hoc analysis; *P < 0.05, **P < 0.01, vehicle *vs*. MPEP-treated group). N = 3 animals per group.

To test the hypothesis that the activity of P2X_3_ in astrocytes is regulated by mGluR5, we analyzed the effect of MPEP on air-puff thresholds and P2X_3_ expression in astrocytes in CCI-ION rats (Figs 7 and 8). Intracisternal administration of MPEP (0.01, 0.1 ng) attenuated mechanical allodynia produced by CCI-ION, compared to vehicle treatment (Fig. 8a): Air-puff thresholds increased significantly 90 min after administration of 0.1 ng MPEP, and this effect lasted 240 min. Also, following MPEP treatment, the gold particle density for P2X_3_ in the fine and terminal astrocytic processes in CCI-ION rats was not increased as in CCI-ION rats treated with vehicle (Figs 7 and 8b). These findings suggest that mGluR5 may mediate the increase of P2X_3_ in astrocytic processes in the CCI-ION model.

## Discussion

In this study, we report for the first time that 1) astrocytes in the Vc express P2X_3_ receptor that is implicated in the neuropathic pain_,_ preferentially in their fine-caliber processes 2) the number of fine astrocytic processes and the density of P2X_3_ receptors in these processes are increased following CCI-ION, and 3) the increased expression of P2X_3_ in fine astrocytic processes is abolished, and the mechanical allodynia is alleviated in the CCI-ION rats following treatment with MPEP, a specific mGluR5 antagonist. These findings suggest the existence of a novel astrocytic P2X_3_-mediated mechanism for craniofacial neuropathic pain that is regulated by mGluR5, especially in the fine astrocytic processes.

That the expression of P2X_3_ in the Vc, measured by the intensity of immunostaining and by the protein level, was increased, and that the administration of A-317491 attenuated the mechanical allodynia following CCI-ION in a dose-dependent manner, are both consistent with previous studies reporting implication of central P2X_3_ receptors in the pathologic pain^4–6^. These results support the notion that P2X_3_ in primary afferent terminals plays a role in pathological pain, and were thus not unexpected. However, we also found P2X_3_ receptors in Vc astrocytes, and have shown that their expression increases following CCI-ION. Based on this, and on further evidence presented below, we propose that craniofacial pathologic pain in the Vc is at least in part mediated by P2X_3_ in astrocytes.

Fine astrocytic processes, which are spatially related to synapses and their associated neuropil, preferentially express a variety of neurotransmitter receptors and mediators, suggesting that this is the site where astrocytes exert their regulatory role in modulating the synaptic transmission^15,16,21,23^. Under normal conditions, these processes exhibit transient structural plasticity (extension and retraction), and thus continuously modify their association with nearby synapses and are involved in the regulation of synaptic transmission^19,21,24,25^. For example, in the hypothalamus of lactating rats, astrocytic processes retract from the synapse, which reduces glutamate clearance from synapses^18,26^. Extension of astrocytic processes has also been observed in the hippocampus after LTP induction^27,28^. The increased number of fine astrocytic processes, but not soma and large processes, following CCI-ION in the present study may be a result of the same kind of structural plasticity. Alternatively, there may be a new growth of fine astrocytic processes following CCI-ION, perhaps resulting in an increase of the overall area of apposition to synapses and the associated neuropile. Gold particle density for P2X_3_ was far lower in astrocytic soma and large process than in fine process in control rats. In addition, the expression of P2X_3_ receptors, particularly those inserted in the plasma membrane, was significantly increased in fine astrocytic processes, but not in soma and large process, following CCI-ION. This may indicate that synthesis and transport of astrocytic P2X_3_ is increased following CCI-ION and the P2X_3_, immediately after its synthesis in the soma, is transported to plasma membrane of astrocytic fine process where it may function. It may be another way to increase the sensitivity of astrocytes to ATP released by neurons, especially in their fine processes.

The hypertrophy of the cell soma and processes, and upregulation of GFAP are characteristic of reactive astrocytes following nerve injury and inflammation^13,14,29^. GFAP is a cytoskeletal protein so this effect may be explained by the need for stronger structural support for the increased number of fine astrocytic process in pathologic conditions including CCI-ION. At present, what causes formation of new fine astrocytic processes following CCI-ION remains unknown. If the ultimate consequence of these cell transformations is modulation of glutamatergic transmission in neuronal circuits, it is reasonable to suppose that the signal for inducing reactive morphology in astrocytes is glutamate itself. This is consistent with the observations that application of glutamate in cultured astrocytes induces formation and extension of filopodia^30–32^ and that conversion of Ca^2+^-permeable into Ca^2+^-impermeable AMPA glutamate receptors in Bergmann glial cells in the cerebellum induces retardation of fine astrocytic processes, which impairs glutamate reuptake from neuronal synapses^33^.

mGluR5, an important component of the system of neuron-glia interactions^34^, plays a crucial role in the development of pathologic pain following nerve injury^35–37^. It is expressed preferentially in fine astrocytic processes^32,38^ and is upregulated in reactive astrocytes^39^. In the present study, the upregulation of P2X_3_ in fine astrocytic processes was abolished, and the mechanical allodynia was alleviated, in CCI-ION rats following intracisternal administration of the mGluR5 antagonist MPEP, suggesting that P2X_3_ expression may be regulated by astrocytic mGluR5, and that the anti-allodynic effect of MPEP may be mediated by P2X_3_. The relation between mGluR5 inhibition and P2X_3_ expression can be also supported by the analysis showing that drug effect onset is different between A-317491 and MPEP (p < 0.05): Thus, A-317491 attenuated mechanical allodynia in 15 min after intracisternal administration, while MPEP attenuated in 60 min (data not shown in the Results). These results may be due to the different mechanisms of the drugs: A-317491 attenuates mechanical allodynia by direct inhibition of astrocytic P2X_3_, while MPEP attenuates mechanical allodynia by indirect regulation of astrocytic P2X_3_ in rats with CCI-ION. The upregulation of astrocytic P2X_3_ following CCI-ION may be driven by activation of astrocytic mGluR5 in response to the excess glutamate released from terminals of primary afferent neurons or from astrocytes following nerve injury^40,41^. One mechanism for that is suggested by the observations that activation of mGluR5 in astrocytes induces phosphorylation of ERK2^42,43^, which is important in neuropathic pain and in the regulaton of P2X_3_ expression^44^. The present study only showed inhibition of astrocytic P2X_3_ upregulation by mGluR5 inhibition in CCI-ION rats. Further experiment showing change in astrocytic P2X_3_ expression by mGluR5 activation may be also needed to ensure the assumption of astrocytic P2X_3_ regulation by mGluR5. Antinociceptive effect produced by intracisternal administration of A-317491 and MPEP shown in the present study might not be specifically mediated by astrocytes. It also could be mediated by various types of neurons and glial cells that express P2X_3_ and mGluR5 in the brainstem.

## Methods

### Animals and tissue preparation

All experiments were conducted in observance of the guidelines of National Institutes of Health, and under approval by the Kyungpook National University Intramural Animal Care and Use Committee. Seventy two male Sprague-Dawley rats (290–310 g) were used for this study: Twenty eight rats were used for a behavioral assay for mechanical allodynia, following CCI-ION, 6 rats for LM immunohistochemistry, 8 rats for Western blot analysis after CCI-ION, 6 rats for EM immunocytochemistry 7 days after CCI-ION, 18 rats for behavioral assay testing for the effect of MPEP on the mechanical allodynia in CCI-ION rats, 6 rats for EM immunohistochemistry 2 hrs after MPEP treatment 7 days post CCI-ION.

For immunohistochemistry, the rats were anesthetized with an intraperitoneal injection of 80 mg/kg sodium pentobarbital and perfused through the heart with 100 ml of heparinized 0.9% saline, followed by 500 ml of freshly-prepared 4% paraformaldehyde in phosphate buffer (PB: 0.1 M, pH 7.4), with (for EM) or without (for LM) 0.01% glutaraldehyde. The brainstem was dissected, fixed in the fixative used for perfusion for an additional 2 hours at 4 °C, and immersed in 30% sucrose in PB overnight. For LM, sections were cut at 40 μm on a freezing microtome, and for EM, on a Vibratome at 50 μm.

### Immunohistochemistry

For immunofluorescent staining, sections were incubated with 50% ethyl alcohol for 30 minutes, with 10% normal donkey serum (NDS; Jackson ImmunoResearch) for 30 minutes, and with rabbit anti-P2X_3_ antibody (1:1,000; APR-016, Alomone) in phosphate-buffered saline (PBS; 0.01 M, pH 7.4) overnight. After incubation in PBS for 3 × 5 minutes, and with 2% NDS for 10 minutes, sections were transferred to Cy3-conjugated donkey anti-rabbit antibody (1:200; 711-165-152, Jackson ImmunoResearch) in PBS for 2 hours. After that, sections were rinsed in PBS and mounted with Vectashield (Vector Laboratories). Micrographs were obtained using Zeiss Axioplan 2 microscope (Carl Zeiss) with an attached Ex*i* digital camera (Q-imaging Inc.).

Quantitative analysis of P2X_3_ expression in Vc was performed on six randomly-selected sections from each of three rats with CCI-ION and three sham-operated rats. The superficial lamina of the middle part of the Vc (where the maxillary branch of the trigeminal nerve terminates^45^) was analyzed using ImageJ (NIH). The threshold for considering each pixel “immunopositive” was set at 120 (all images had 256 gray levels). The percent area within a 100 × 100 μm square that had immunostaining, and the ratio of labeling intensity over background were presented as mean ± standard error of mean (SEM). The difference in mean percent area that is immunopositive and the intensity of immunostaining was compared between groups using unpaired Student’s t-test.

For EM immunohistochemistry, sections were frozen on dry ice for 20 minutes, then thawed in PBS, and incubated with 1% sodium borohydride for 30 minutes, with 3% H_2_O_2_ for 10 minutes, and with 10% NDS for 30 minutes. Sections were transferred to a mixture of mouse anti-GFAP (1:5,000; MAB360, Chemicon) and rabbit anti-P2X_3_ (1:200; APR-016, Alomone) antibodies in PBS overnight at 4 °C. Sections were incubated in PBS for 3 × 15 minutes, and in a mixture of biotinylated donkey anti-mouse antibody (1:200; 715-065-150, Jackson ImmunoResearch) and donkey anti-rabbit antibody conjugated to 1 nm gold (1:50; #25700, Jackson ImmunoResearch) for 1–3 hours. Sections were further incubated with 1% glutaraldehyde for 10 minutes, with PBS 3 × 15 minutes, with IntenSE^TM^ M silver intensification solution (Amersham) for 4 minutes, and with 0.1 M sodium acetate and PB for 10 minutes. Further, sections were incubated with ExtrAvidin peroxidase (1:5,000; Sigma) for 1 hour, and with nickel-intensified 3,3′-diaminobenzidine tetrahydrochloride (Ni-DAB). Sections were rinsed in PB, treated with OsO_4_, dehydrated in ethanol, and flat-embedded in Durcupan ACM (Fluka). Wafer-embedded sections were cured at 60 °C for 48 hrs. Chips containing the superficial lamina of the middle part of Vc were cut out of the wafers and mounted onto Durcupan cylinders. Thin sections were cut at a 70 nm thickness with a diamond knife onto single slot Ni grids, coated in advance with formvar, and counterstained with uranyl acetate and lead citrate. Grids were examined at 80 kV accelerating voltage on a Hitachi H-7500 electron microscope and photomicrographs were collected with a SC1000 CCD camera (Gatan) running DigitalMicrograph software.

To test antibody specificity, sections were treated according to the above protocols, except that primary and secondary antibodies were omitted, which abolished specific staining. We also looked for consistency of immunolabeling in adjacent thin sections of the same astrocytic process or axon terminal to confirm the selectivity of immunostaining. The immunostaining for P2X_3_ in the Vc was similar to what we reported previously^9^. The immunostaining for P2X_3_ was also eliminated by preadsorption with 4.8 µg/nl of the antigenic peptide (APR016AG0440, Alomone).

For quantitative analysis, the GFAP+ and P2X_3_+/GFAP+ astrocytes and astrocytic processes was counted, and the diameter of GFAP+ astrocytes was measured in electron micrographs. Ninety electron micrographs at 25,000 × were taken of all GFAP+ astrocytes within 3,500 μm^2^ from 2 thin sections in each of three CCI-ION and three sham-operated rats, and from 2 thin sections in each of three MPEP-treated and three vehicle-treated rats following CCI-ION. We divided the GFAP+ astrocytic processes into three groups: Large (>1 μm in diameter), fine (<1 μm in diameter), and terminal (<0.3 μm in diameter). We also categorized the gold particles coding for P2X_3_ in the GFAP+ astrocytes and axon terminal into two groups: Membrane-bound (at the plasma membrane and within 25 nm from it) and cytoplasmic (>25 nm away from the plasma membrane). The density of immunolabeling for P2X_3_ was determined by manual counting of gold particles over the area of GFAP+ profiles and axon terminals; cell nuclei and mitochondria were excluded. The between-group differences in the number of GFAP + and P2X_3_+/GFAP+ astrocytes, and gold particle density were compared using unpaired Student’s *t*-test.

### Western blot

Rats were perfused with saline and the brainstem was dissected out and blocked to include Vc. All chemicals, unless stated otherwise, were purchased from Sigma-Aldrich. The samples were homogenized in extraction buffer (in mM; 20 Tris-HCl pH 7.4, 5 EDTA, 140 NaCl, 1 PMSF, 1 Na_3_VO_4_, 10 NaF, 1% Triton X-100, and 1 mg/ml aprotinin) at 4 °C. The extracts were centrifuged at 12,000 g for 20 minutes at 4 °C. Proteins in supernatant were measured with Bio-Rad Protein Assay kit (Bio-Rad), denatured at 95 °C for 5 minutes with SDS-loading buffer, separated by electrophoresis on SDS-PAGE gel, and transferred to Immobilon-P membranes (EMD Millipore). The membranes were incubated with blocking solution (TBS, 5% nonfat milk, 0.02% NaN_3_) for 2 hours and incubated with mouse anti-β-actin (1:2,000; #sc81178, Santa Cruz) and rabbit anti-P2X_3_ (1:1,000; APR-016, Alomone) antibodies overnight at 4 °C. The membranes were washed with TBS and incubated with goat anti-mouse IgG (1:2,000; #sc2005, Santa Cruz) or goat anti-rabbit IgG (1:2000; #sc2004, Santa Cruz) antibodies for 1 hour at room temperature, then treated with ECL solution (EMD Millipore) and exposed on an autoradiography film (Agfa,). Differences in protein concentrations between groups (n = 4 for each CCI-ION and sham groups) were compared using unpaired Student’s *t*-test. Data are presented as the mean ± SEM.

### Behavioral assays

Rats were anesthetized with 40 mg/kg ketamine and 4 mg/kg xylazine, and CCI-ION was performed following the original description^46^. A 1 cm incision was made along the gingivo-buccal margin, proximal to the first molar. Approximately 0.5 cm of the infraorbital nerve was freed up from the surrounding tissue, and two 5-0 chromic gut ligatures were tied loosely around it. The incision was closed with two 4-0 silk sutures. The sham operation was identical, except for the ligation of the infraorbital nerve.

On postoperative day 4, rats were re-anesthetized, mounted on a stereotaxic frame, and a PE 10 polyethylene tubing was inserted through a small hole in the atlanto-occipital membrane and dura using a 27-gauge needle, as described previously^47,48^. The tip of the cannula was placed dorsal to the obex. The polyethylene tube was led subcutaneously to the top of the head and secured in place using a stainless steel screw and dental acrylic resin. A recovery period of 72 hours was allowed.

Rats were habituated for at least 30 min to a customized cage in a darkened and noise-free room. Withdrawal response was measured after 10 trials of constant air-puff pressure (4-second duration and 10-second intervals), as described previously^49,50^; the response threshold was determined as the air-puff pressure at which the rat responded in 50% of the trials. The cut-off pressure was 40 psi.

To evaluate the effect of A-317491 on mechanical allodynia, we examined changes in air-puff threshold after intracisternal administration of 1 μg, 5 μg/10 μl of A-317491 on post-operative day 7. We also examined the effect of MPEP, a specific mGluR5 antagonist, on mechanical allodynia. After intracisternal administration of 0.01, 0.1 ng/10 μl MPEP on post-operative day 7, the withdrawal behaviors to the air-puff were measured. A-317491 and MPEP were purchased from Sigma-Aldrich.

Differences between groups were compared using repeated-measures analysis of variance, followed by Holm-Sidak post-hoc analysis. Data are presented as the mean ± SEM.

### Data Availability

The datasets generated during and/or analysed during the current study are available from the corresponding author on reasonable request.

## References

[1] Chen CC (1995). A P2X purinoceptor expressed by a subset of sensory neurons. Nature.

[2] Chizh BA, Illes P (2001). P2X Receptors and Nociception. Pharmacol. Rev..

[3] Dunn PM, Zhong Y, Burnstock G (2001). P2X receptors in peripheral neurons. Prog. Neurobiol..

[4] Barclay J (2002). Functional downregulation of P2X3 receptor subunit in rat sensory neurons reveals a significant role in chronic neuropathic and inflammatory pain. J. Neurosci..

[5] Honore P (2002). Analgesic profile of intrathecal P2X3 antisense oligonucleotide treatment in chronic inflammatory and neuropathic pain states in rats. Pain.

[6] McGaraughty S (2003). Effects of A-317491, a novel and selective P2X3/P2X2/3 receptor antagonist, on neuropathic, inflammatory and chemogenic nociception following intrathecal and intraplantar administration. Br. J. Pharmacol..

[7] Ambalavanar R, Moritani M, Dessem D (2005). Trigeminal P2X3 receptor expression differs from dorsal root ganglion and is modulated by deep tissue inflammation. Pain.

[8] Bradbury EJ, Burnstock G, McMahon SB (1998). The expression of P2X3 purinoreceptors in sensory neurons: effects of axotomy and glial-derived neurotrophic factor. Mol. Cell. Neurosci..

[9] Kim YS (2008). Expression of P2X3 receptor in the trigeminal sensory nuclei of the rat. J. Comp. Neurol..

[10] Vulchanova L (1998). P2X3 is expressed by DRG neurons that terminate in inner lamina II. Eur. J. Neurosci..

[11] Chiang CY, Li Z, Dostrovsky JO, Hu JW, Sessle BJ (2008). Glutamine uptake contributes to central sensitization in the medullary dorsal horn. Neuroreport.

[12] Chiang C-Y (2007). Astroglial glutamate-glutamine shuttle is involved in central sensitization of nociceptive neurons in rat medullary dorsal horn. J. Neurosci..

[13] Okada-Ogawa A (2009). Astroglia in medullary dorsal horn (trigeminal spinal subnucleus caudalis) are involved in trigeminal neuropathic pain mechanisms. J. Neurosci..

[14] Tsuboi Y (2011). Modulation of astroglial glutamine synthetase activity affects nociceptive behaviour and central sensitization of medullary dorsal horn nociceptive neurons in a rat model of chronic pulpitis. Eur. J. Neurosci..

[15] Bezzi P (2004). Astrocytes contain a vesicular compartment that is competent for regulated exocytosis of glutamate. Nat. Neurosci..

[16] Verkhratsky A, Orkand RK, Kettenmann H (1998). Glial calcium: homeostasis and signaling function. Physiol. Rev..

[17] Grosche J (1999). Microdomains for neuron-glia interaction: parallel fiber signaling to Bergmann glial cells. Nat. Neurosci..

[18] Oliet SH, Piet R, Poulain DA (2001). Control of glutamate clearance and synaptic efficacy by glial coverage of neurons. Science.

[19] Hirrlinger J, Hülsmann S, Kirchhoff F (2004). Astroglial processes show spontaneous motility at active synaptic terminals *in situ*. Eur. J. Neurosci..

[20] Sun D, Jakobs TC (2012). Structural remodeling of astrocytes in the injured CNS. Neuroscientist.

[21] Theodosis DT, Poulain DA, Oliet SHR (2008). Activity-dependent structural and functional plasticity of astrocyte-neuron interactions. Physiol. Rev..

[22] Won KA (2014). The glial-neuronal GRK2 pathway participates in the development of trigeminal neuropathic pain in rats. J. Pain.

[23] Reichenbach A, Derouiche A, Kirchhoff F (2010). Morphology and dynamics of perisynaptic glia. Brain Res. Rev..

[24] Haber M, Zhou L, Murai KK (2006). Cooperative astrocyte and dendritic spine dynamics at hippocampal excitatory synapses. J. Neurosci..

[25] Nishida H, Okabe S (2007). [Visualization of synapse-glia dynamics]. Brain nerve = Shinkei kenkyū no shinpo.

[26] Piet R, Vargová L, Syková E, Poulain DA, Oliet SHR (2004). Physiological contribution of the astrocytic environment of neurons to intersynaptic crosstalk. Proc. Natl. Acad. Sci. USA.

[27] Lushnikova I, Skibo G, Muller D, Nikonenko I (2009). Synaptic potentiation induces increased glial coverage of excitatory synapses in CA1 hippocampus. Hippocampus.

[28] Wenzel J, Lammert G, Meyer U, Krug M (1991). The influence of long-term potentiation on the spatial relationship between astrocyte processes and potentiated synapses in the dentate gyrus neuropil of rat brain. Brain Res..

[29] Cady RJ, Glenn JR, Smith KM, Durham PL (2011). Calcitonin gene-related peptide promotes cellular changes in trigeminal neurons and glia implicated in peripheral and central sensitization. Mol. Pain.

[30] Cornell-Bell AH, Thomas PG, Caffrey JM (1992). Ca2+ and filopodial responses to glutamate in cultured astrocytes and neurons. Can. J. Physiol. Pharmacol..

[31] Cornell-Bell AH, Thomas PG, Smith SJ (1990). The excitatory neurotransmitter glutamate causes filopodia formation in cultured hippocampal astrocytes. Glia.

[32] Lavialle M (2011). Structural plasticity of perisynaptic astrocyte processes involves ezrin and metabotropic glutamate receptors. Proc. Natl. Acad. Sci. USA.

[33] Iino M (2001). Glia-synapse interaction through Ca2+ -permeable AMPA receptors in Bergmann glia. Science.

[34] Panatier A, Robitaille R (2016). Astrocytic mGluR5 and the tripartite synapse. Neuroscience.

[35] Dogrul A, Ossipov MH, Lai J, Malan TP, Porreca F (2000). Peripheral and spinal antihyperalgesic activity of SIB-1757, a metabotropic glutamate receptor (mGLUR5) antagonist, in experimental neuropathic pain in rats. Neurosci. Lett..

[36] Hudson LJ (2002). Metabotropic glutamate receptor 5 upregulation in A-fibers after spinal nerve injury: 2-methyl-6-(phenylethynyl)-pyridine (MPEP) reverses the induced thermal hyperalgesia. J. Neurosci..

[37] Varty GB (2005). The antinociceptive and anxiolytic-like effects of the metabotropic glutamate receptor 5 (mGluR5) antagonists, MPEP and MTEP, and the mGluR1 antagonist, LY456236, in rodents: a comparison of efficacy and side-effect profiles. Psychopharmacology (Berl)..

[38] Panatier A (2011). Astrocytes are endogenous regulators of basal transmission at central synapses. Cell.

[39] Gwak YS, Hulsebosch CE (2005). Upregulation of Group I metabotropic glutamate receptors in neurons and astrocytes in the dorsal horn following spinal cord injury. Exp. Neurol..

[40] Coderre TJ, Kumar N, Lefebvre CD, Yu JSC (2005). Evidence that gabapentin reduces neuropathic pain by inhibiting the spinal release of glutamate. J. Neurochem..

[41] Yan X, Jiang E, Gao M, Weng H-R (2013). Endogenous activation of presynaptic NMDA receptors enhances glutamate release from the primary afferents in the spinal dorsal horn in a rat model of neuropathic pain. J. Physiol..

[42] Peavy RD, Conn PJ (1998). Phosphorylation of mitogen-activated protein kinase in cultured rat cortical glia by stimulation of metabotropic glutamate receptors. J. Neurochem..

[43] Peavy RD, Chang MSS, Sanders-Bush E, Conn PJ (2001). Metabotropic Glutamate Receptor 5-Induced Phosphorylation of Extracellular Signal-Regulated Kinase in Astrocytes Depends on Transactivation of the Epidermal Growth Factor Receptor. J. Neurosci..

[44] Yu J, Zhao C, Luo X (2013). The effects of electroacupuncture on the extracellular signal-regulated kinase 1/2/P2X3 signal pathway in the spinal cord of rats with chronic constriction injury. Anesth. Analg..

[45] Takemura M, Sugiyo S, Moritani M, Kobayashi M, Yonehara N (2006). Mechanisms of orofacial pain control in the central nervous system. Arch. Histol. Cytol..

[46] Imamura Y, Kawamoto H, Nakanishi O (1997). Characterization of heat-hyperalgesia in an experimental trigeminal neuropathy in rats. Exp. brain Res..

[47] Yaksh TL, Rudy TA (1976). Chronic catheterization of the spinal subarachnoid space. Physiol. Behav..

[48] Yang KY (2015). Blockade of spinal glutamate recycling produces paradoxical antinociception in rats with orofacial inflammatory pain. Prog. Neuropsychopharmacol. Biol. Psychiatry.

[49] Ahn DK (2009). Compression of the trigeminal ganglion produces prolonged nociceptive behavior in rats. Eur. J. Pain.

[50] Ahn DK (2009). Intratrigeminal ganglionic injection of LPA causes neuropathic pain-like behavior and demyelination in rats. Pain.

